# Cardiac‐specific Mst1 deficiency inhibits ROS‐mediated JNK signalling to alleviate Ang II‐induced cardiomyocyte apoptosis

**DOI:** 10.1111/jcmm.13958

**Published:** 2018-10-19

**Authors:** Zheng Cheng, Mingming Zhang, Jianqiang Hu, Jie Lin, Xinyu Feng, Shanjie Wang, Tingting Wang, Erhe Gao, Haichang Wang, Dongdong Sun

**Affiliations:** ^1^ Department of Cardiology Tangdu Hospital Fourth Military Medical University Xi'an China; ^2^ Department of Cardiology Xijing Hospital Fourth Military Medical University Xi'an China; ^3^ Center for Translational Medicine Temple University School of Medicine Philadelphia Pennsylvania

**Keywords:** Angiotensin II, apoptosis, ASK1, JNK, Mst1, ROS, Trx

## Abstract

Apoptosis is associated with various myocardial diseases. Angiotensin II (Ang II) plays a central role in the pathogenesis of RAAS‐triggered cardiac apoptosis. Our previous studies showed that mammalian Ste20‐like kinase 1 (Mst1) aggravates cardiac dysfunction in cardiomyocyte under pathological conditions, but its role in Ang II‐mediated cardiomyocyte apoptosis is not known. We addressed this in the present study by investigating whether cardiac‐specific Mst1 knockout can alleviate Ang II‐induced cardiomyocyte apoptosis along with the underlying mechanisms. In vitro and in vivo experiments showed that Ang II increased intracellular reactive oxygen species (ROS) production and cardiomyocyte apoptosis; these were reversed by administration of the ROS scavenger N‐acetylcysteine and by Mst1 deficiency, which suppressed c‐Jun N‐terminal kinase (JNK) phosphorylation and downstream signaling. Interestingly, Mst1 knockout failed to alleviate Ang II‐induced phosphorylation of extracellular signal‐regulated kinase 1/2, and inactivated apoptosis signal‐regulating kinase1 (ASK1) by promoting its association with thioredoxin (Trx), which reversed the Ang II‐induced activation of the ASK1–JNK pathway and suppressed Ang II‐induced cardiomyocyte apoptosis. Thus, cardiac‐specific Mst1 knockout inhibits ROS‐mediated JNK signalling to block Ang II‐induced cardiomyocyte apoptosis, suggesting Mst1 as a potential therapeutic target for treatment of RAAS‐activated heart failure.

## 1 INTRODUCTION

Heart failure (HF) is a condition in which the heart cannot supply the body's tissues with sufficient blood, resulting in a cascade of changes that lead to severe fatigue, breathlessness, and death.^1^ Hyperactivation of the renin‐angiotensin‐aldosterone system (RAAS) is known to occur in HF and is thought to trigger cardiomyocyte apoptosis, which is mediated by angiotensin II (Ang II).^2^^,^ 3 In clinics and hospitals, various strategies have been used to counteract the action of Ang II with limited success.^4^

Reactive oxygen species (ROS) generated during cellular aerobic respiration and metabolism have been implicated in cardiovascular pathologies.^5^ The production of large amount of ROS by action of Ang II can trigger the destruction of structures such as intracellular proteins, organelle, and DNA.^6^, ^7^, ^8^ Attenuating ROS levels and thereby reducing oxidative stress can alleviate cardiomyocyte apoptosis.^5^, ^9^

Mitogen‐activated protein kinases (MAPKs) are a conserved family of serine/threonine protein kinases that are involved in many physiological and pathological processes.^10^, ^11^ MAPKs include extracellular signal‐regulated kinase (ERK) 1/2, c‐Jun N‐terminal kinase (JNK), and P38. Inhibition of JNK and ERK1/2 has been shown to block cardiac apoptosis.^12^ Apoptosis signal‐regulating kinase 1 (ASK1), a MAPK kinase kinase that is activated by various types of pathological stimuli including Ang II‐induced oxidative stress, can activate JNK signalling to induce cell apoptosis.^13^, ^14^, ^15^, ^16^ Thioredoxin (Trx) is a redox regulatory protein that inhibits the kinase activity of ASK1 by directly binding to its N terminus.^15^, ^17^ ROS production induced by Ang II results in the dissociation of ASK1 from Trx and sequent apoptosis via JNK signalling. Thus, inhibiting ASK1 activity by targeting Trx is a potential therapeutic strategy to counteract Ang II‐triggered cardiomyocyte apoptosis.

Mammalian Ste20‐like kinase (Mst) 1 is a serine‐threonine kinase and a component of the Hippo signalling pathway that has been shown to promote apoptosis and induce HF.^18^ Our previous studies suggested a potential role for Mst1 in regulating cardiomyocyte autophagy; moreover, Mst1 knockout protected cardiomyocytes against apoptosis in diabetic cardiomyopathy.^19^, ^20^ However, the effect of cardiac‐specific Mst1 knockout (Mst1^Δ/Δ^) in Ang II‐induced cardiac apoptosis and the underlying mechanisms are not well understood.

We addressed this in the present study by investigating whether cardiac‐specific Mst1 knockout can alleviate Ang II‐induced cardiomyocyte apoptosis. Furthermore, we tested the underlying mechanisms that Mst1‐specific knockout effectively counteracted Ang II‐induced dissociation of Trx from ASK1, thus inhibiting ASK1/JNK signalling‐mediated activation of cardiomyocyte apoptosis.

## 2 MATERIALS AND METHODS

For methods, materials and ethics statement in detail, please refer to the electronic supplementary material (ESM) Methods.

### 2.1 Cardiomyocyte‐specific Mst1 knockout mice

Cardiomyocyte‐specific Mst1 knockout (Mst1^Δ/Δ^) mice were generated by standard Cre‐LoxP‐based gene targeting strategies. The final targeting vector (LoxP sites are A12242 to T12275 and A6695 to T6728; Exons are G13681 to A13724, T13561 to T13620, C7270 to G7320 and G4350 to G4478) for Mst1‐conditional knockout was constructed and subsequently delivered to ES cells (C57BL/6 background). Then some clones were selected for blastocyst microinjection, followed by chimera production. Founders were confirmed as germline‐transmitted via crossbreeding with wild‐type (Cyagen Biosciences Inc, Suzhou). After that Neo delete F1 heterozygous mutant mice (Mst1^flox/+^, three male and four female) were confirmed. F2 were generated by F1 and Mst1^flox/flox^ (Mst1^fl/fl^) mice were identified from F2 by PCR screening. Next, we crossed Mst1^fl/fl^ mice with αMHC‐MerCreMer Mice (Jackson Laboratories, USA) (C57BL/6 background) as F3 (αMHC‐MerCreMer: Mst1^flox/+^). By repeatedly crossing F3 with Mst1^flox/flox^ mice, F4 were generated. Then, we distinguished Mst1^flox/flox^ mice from F4 and indentified the αMHC‐MerCreMer:Mst1^flox/flox^ (Mst1^Δ/Δ^) mice from Mst1^fl/fl^ mice of F4 by PCR screening. Tamoxifen (Sigma Aldrich, 40 mg/kg) in corn oil was administered ip in 6‐week‐old male Mst1^Δ/Δ^ mice for 5 days in succession.^21^ To all mice after the last tamoxifen injection, we allowed 7 weeks recovery before any experiments because expression of Cre recombinase in heart can induce a transient cardiomyopathy that dissipates 5 weeks after tamoxifen‐induced Cre expression.^21^ Finally, we confirmed the effectiveness of Cre recombinase in adult cardiomyocytes by PCR screening and Western blotting before setting up animal model. See ESM Methods and ESM Figure S1 to S2 for details.

### 2.2 Animal model

Mice were anaesthetized with 2% isoflurane, and a mini‐osmotic pump (Alzet osmotic pump; BT‐258, 2006) was implanted subcutaneously between the scapulae. In Ang II–treated groups, pumps were infused with Ang II (Merck1656, 1000 ng/kg per/min) for 46 days. Control mice received infusion of comparable volume saline. Mst1^fl/fl^ and Mst1^Δ/Δ^ mice were randomly assigned to control groups or Ang II groups. All groups had similar hemodynamic indexes at baseline. The mice were divided into the following groups: (a) Mst1^fl/fl^; (b) Mst1^Δ/Δ^; (c) Ang II+Mst1^fl/fl^; (d) Ang II+Mst1^Δ/Δ^ (n = 9 mice per group). 46 days later, all mice were executed in order to analyse the development of myocardial apoptosis and check the activation of corresponding signalling pathways. Furthermore, to verify whether the protective role of Mst1 conditional knockout mediated via eliminating ROS, the new trial was carried out by intervention with NAC, the ROS scavenger. See ESM methods for details.

### 2.3 Mean arterial pressure

Mean arterial pressure (MAP) was measured by non‐invasive tail cuff system (Softron BP‐2010A, China) every 2 days.

### 2.4 Echocardiography

Echocardiography was conducted in M‐mode using an echocardiography system with a 15 MHz linear transducer (Vevo 2100, Canada) before execution.^20^

### 2.5 Histological analyses

Heart specimens were fixed with formalin, embedded in paraffin, and sectioned at 6 μm thickness as previously described.^19^, ^20^

### 2.6 Measurement of tissue superoxide

Part of left ventricle myocardium were immediately frozen in OCT embedding agent (Sakura, 4583), and these tissues were cut into 8 μm frozen sections by freezing microtome (Thermo, Cryotome E). Three sections per left ventricle were selected randomly. Sections were incubated with fluorescent probe DHE (5 mmol/L Beyotime S0063, China) for 30 minutes at 37°C away from light. DAPI (Solarbio, China) was used for nucleic acid staining. The fluorescence intensity of DHE evaluated by fluorescence microscope (Olympus FV1000, Japan) reflects the superoxide levels of tissue in situ.

### 2.7 TUNEL assay of each group

Each groups were fixed in 4% formaldehyde and paraffin embedded, serially cut into 6 μm sections. These sections were stained according to the instructions of the kit (In Situ Cell Death Detection Kit, Roche, Germany).^20^

### 2.8 Immunohistochemistry of p‐JNK

Immunohistochemistry was performed in paraffin sections using a mircowave‐based antigen retrieval method. Antibodies used in this study included: p‐JNK (#4668; Cell Signaling Technology) with 1:100 dilution and an irrelevant isotype rabbit (negative control). For details and statistical methods, see ESM Methods.

### 2.9 Primary neonatal mouse ventricular cardiomyocytes culture and treatment

Primary cultures of neonatal ventricular cardiomyocytes were prepared from 1 to 3‐day‐old wild‐type C57BL/6 mice as previously described.^19^, ^20^ Cardiomyocytes were randomly allocated into the following groups: (a) Control; (b) Control+Ad‐LacZ; (c) Control+Ad‐sh‐Mst1; (d) Ang II; (e) Ang II+Ad‐LacZ; (f) Ang II+Ad‐sh‐Mst1.

### 2.10 Construction and transduction of Mst1 shRNA adenovirus

Adenoviruses harbouring a short hairpin (sh) RNA directed against Mst1 (Ad‐sh‐Mst1) and harbouring control vectors for Ad‐sh‐Mst1 (Ad‐LacZ) were constructed and then transduced to primary cardiomyocyte successfully. The titers of adenoviruses were 1.26*10^10^ PFU/mL. The multiplicity of infection used was 100:1. The shRNA sequence targeting mouse Mst1 is CCCGTTTGTTAAGAGTGCCAAAGGA. See ESM Methods and ESM Figure S3 for details.

### 2.11 Fluorescence detection of Mst1

Fluorescence microscopic detection of Mst1 (ab51134; Abcam) was conducted according to the manufacturer's instructions.

### 2.12 Measurement of intracellular ROS in vitro

Primary cardiomyocytes cultured on confocal dishes were labelled with DCFH‐DA fluorescent probe (10 μmol/L, Beyotime) at 37°C for 20 minutes. Then, cells were rinsed three times with fresh medium. Next, cells were treated with Hoechst 33342 Nuclear stain (Enzo Life Sciences Inc, ENZ‐51035) for 5 minutes. The fluorescence intensity of DCFH‐DA represents intracellular ROS level.

### 2.13 Analyses of MDA, SOD, T‐AOC, and mitochondrial ATP

Intracellular MDA, SOD, T‐AOC (Nanjing Jiancheng Bioengineering Institute, China) and mitochondrial ATP (Beyotime S0027, China) were measured using commercial assay kits according to manufacturer's Instructions.

### 2.14 Assessment of primary cardiomyocyte apoptosis

As described previously, TUNEL staining was performed with fluorescein‐dUTP (In Situ Cell Death Detection Kit, Roche Diagnostics) for apoptotic cell nuclei and DAPI stained cell nuclei.^20^ Besides, apoptosis was induced by adding H_2_O_2_ (12.5 μM, 100 μM and 800 μM) into 1 mL fresh medium for 4 hours as external positive control.

### 2.15 AO/EB staining

Cardiomyocytes cultured on confocal dishes were treated with 20 μL AO/EB solution (10 μL of AO; 10 μL of EB) (Solarbio, China) and incubated for 5 minutes at 37°C, and then examined by fluorescence microscope.

### 2.16 Detection of JNK nuclear translocation in vitro

Primary antibody JNK (1:300, ab179461, Abcam) was employed to analyse the ratio of JNK nuclear translocation in primary cardiomyocyte. For details, see ESM Methods.

### 2.17 Western blot evaluation

Protein was extracted from myocardial tissues or primary cardiomyocytes. Protein quantitation was modified by Bradford assay (Bio‐Rad Laboratories, Hercules, USA). See ESM Methods for detailed methods and materials.

### 2.18 Detect Trx/ASK1 interaction in myocardium

The co‐immunoprecipitation of Trx and ASK1 was performed following the protocol.^22^, ^23^ See ESM Methods for details.

### 2.19 Co‐localisation of Trx and ASK1

In cardiac muscle, the sequential method of immunofluorescence double stainings was used to assess co‐localization of Trx and ASK1. See ESM Methods for details.

### 2.20 Statistics

The results were analysed and quantified by Image Pro plus 6.0 software. Analyses of differences between groups were carried out using unpaired Student's *t* test, one‐way ANOVA followed by a Fisher's post hoc comparison test. Continuous variables were expressed as mean ± standard error of mean (SEM). Two‐sided tests were used throughout this study, and a *P* value of less than 0.05 was considered as significant. Statistical analyses were done using GraphPad Prism 5.01.

## 3 RESULTS

### 3.1 Mst1 knockout inhibits Ang II‐induced cardiomyocyte apoptosis in vivo

**Figure 1:**
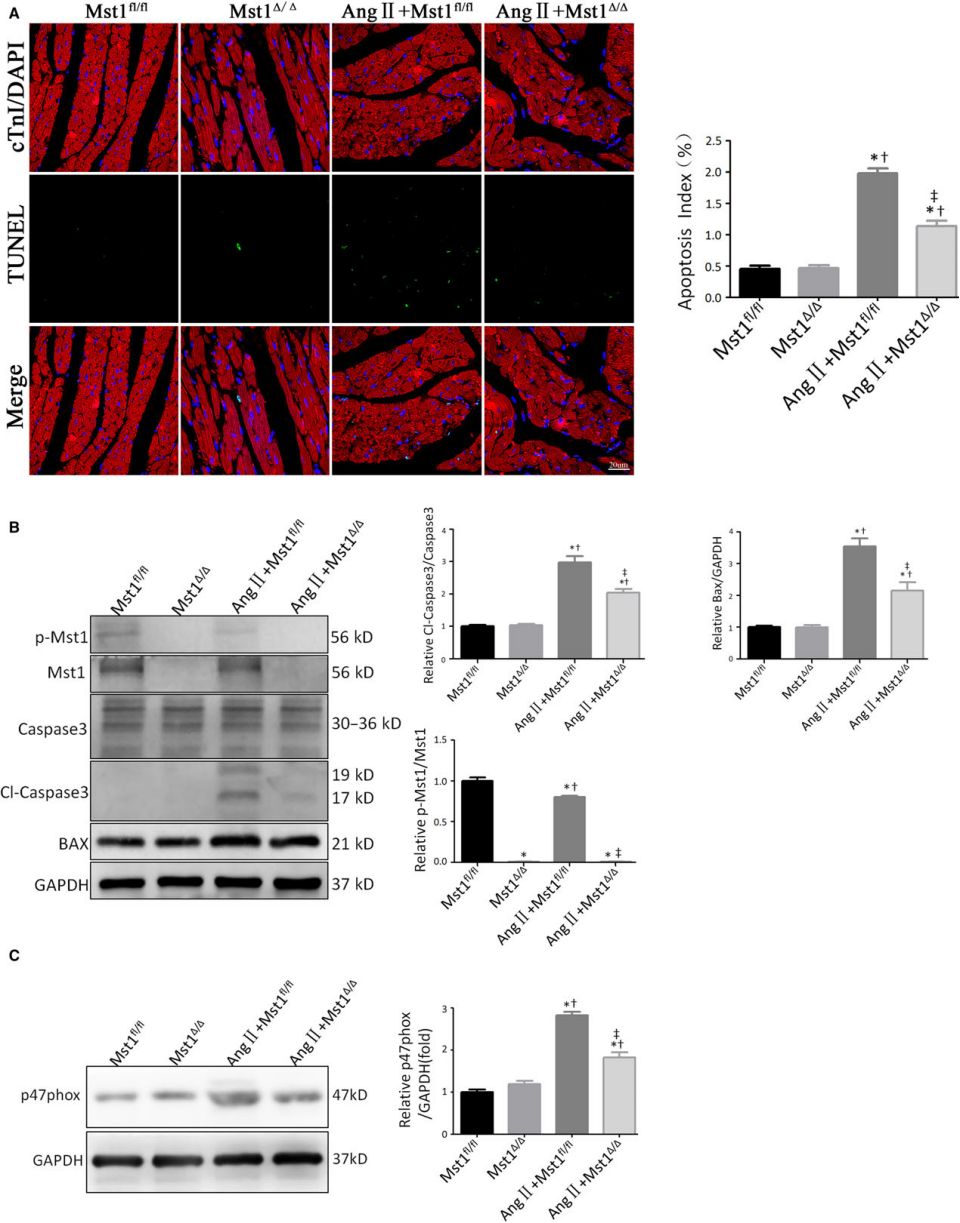
Mst1 knockout inhibits Ang II‐induced cardiomyocyte apoptosis in vivo. A: Representative images of TUNEL assay after 46 days of surgery (n = 9); TUNEL (green), DAPI (blue) and cTroponin I antibody (red). Apoptosis index is expressed as percentage of TUNEL‐positive cardiomyocytes (in green) over total nuclei determined by DAPI staining. Histogram: Apoptosis index (%). B: Immunoblots and quantitative analyses of p‐Mst1/Mst1, Cl‐caspase3/Caspase3 and Bax/GAPDH (n = 9). Histogram: Relative p‐Mst1/Mst1, Cl‐caspase3/Caspase3 and Bax/GAPDH. C: Immunoblots and quantitative analyses of p47^phox^/GAPDH (n = 9). Histogram: Relative intensity of p47^phox^/GAPDH. *P <* *0.05 vs Mst1^fl/fl^ group; ^†^ *P *<* *0.05 vs Mst1^Δ/Δ^ group; ^&ddagger;^ *P *<* *0.05 vs Ang II+Mst1^fl/fl^ group

The terminal deoxynucleotidyl transferase dUTP nick end labelling (TUNEL) assay revealed that Ang II administration aggravated cardiomyocte apoptosis, as evidenced by the increased apoptotic index in Ang II+Mst1^fl/fl^ mice relative to controls (Figure 1A). Importantly, cardiac‐specific Mst1 knockout decreased AngII‐induced myocardial apoptosis relative to Ang II+Mst1^fl/fl^ mice (Figure 1A).

We also evaluated the effect of Mst1 deficiency on the expression of apoptosis‐related proteins by western blotting (Figure 1B). The ratios of cleaved caspase‐3/caspase‐3 and B cell lymphoma 2‐associated X protein (Bax) were higher in the Ang II+Mst1^fl/fl^ group than in control mice (Figure 1B). These results also suggest that Mst1‐specific knockout in the presence of Ang II suppressed apoptosis (Figure 1B).

Moreover, Ang II stimulated the expression of p47^phox^ (Figure 1C), a NADPH oxidase cytosolic subunit that plays an important role in the production of ROS and oxidative stress response.^24^ Interestingly, Mst1 knockout attenuated the protein level of p47^phox^ in mice subjected to chronic Ang II infusion (Figure 1C).

### 3.2 Mst1 deficiency alleviates Ang II‐induced cardiac dysfunction

Chronic Ang II infusion for 46 days resulted in left ventricular (LV) dysfunction (ESM Figure S4A‐D). Cardiomyocyte‐specific Mst1 knockout attenuated this effect, as evidence by an elevated LV ejection fraction (LVEF) and LV fractional shortening (LVFS) as well as decreases in LV end systolic diameter (LVESD) and LV end diastolic diameter (LVEDD) in mice subjected to chronic Ang II infusion (ESM Figure S4A‐D).

### 3.3 Ang II‐induced ROS generation and JNK phosphorylation are suppressed in the absence of Mst1

**Figure 2:**
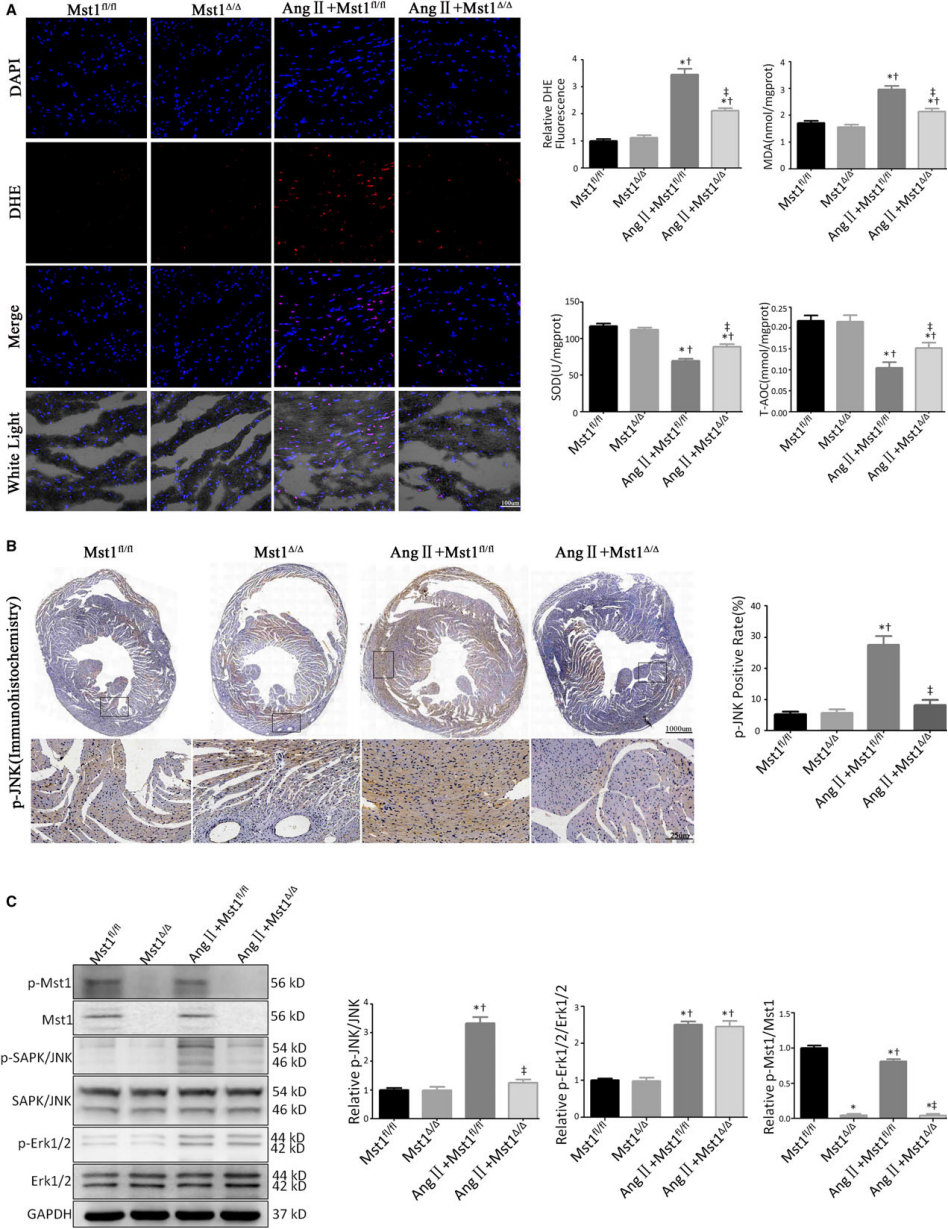
Ang II‐induced ROS generation and JNK phosphorylation are suppressed in the absence of Mst1. A: Measurements of ROS production (n = 9). The effects of ROS generations were measured by the DHE fluorescent probe, the levels of intracellular MDA, SOD, and T‐AOC. Histogram: Relative DHE fluorescence intensity, MDA levels (nmol/mgprot), SOD levels (U/mgprot) and T‐AOC levels (mmol/mgprot). B: Representative images of p‐JNK immunohistochemistry (n = 9). Histogram: p‐JNK positive rate (%). C: Immunoblots and quantitative analyses of p‐Mst1/Mst1, p‐JNK/JNK and p‐Erk1/2/Erk1/2 (n = 9). Histogram: Relative intensity of p‐Mst1/Mst1, p‐JNK/JNK and p‐Erk1/2/Erk1/2. *P <* *0.05 vs Mst1^fl/fl^ group; ^†^ *P *<* *0.05 vs Mst1^Δ/Δ^ group; ^&ddagger;^ *P *<* *0.05 vs Ang II+Mst1^fl/fl^ group

To investigate the effect of cardiomyocyte‐specific Mst1 knockout on ROS production, we used the fluorescent probe dihydroethidium (DHE) to detect superoxide levels in myocardial tissues. Ang II treatment increased DHE fluorescence intensity in myocardial tissues of AngII+Mst1^fl/fl^ mice, an effect that was abrogated by loss of Mst1 (Figure 2A).

We also evaluated the phosphorylation levels of JNK/stress‐activated protein kinase (SAPK) by immunohistochemistry (Figure 2B). Ang II induced JNK phosphorylation in Mst^fl/fl^ mice, while Mst1 deficiency abrogated Ang II‐triggered JNK activation (Figure 2B).

A western blot analysis revealed that Ang II increased the phosphorylation of both JNK and ERK1/2 (Figure 2C). Cardiomyocyte‐specific Mst1 knockout suppressed JNK/SAPK activation (Thr183/Tyr185) in the presence of Ang II, although ERK1/2 phosphorylation was unaffected (Figure 2C).

### 3.4 Mst1 deficiency inhibits ROS‐mediated JNK phosphorylation to attenuate Ang II‐induced cardiomyocyte apoptosis in vivo

**Figure 3:**
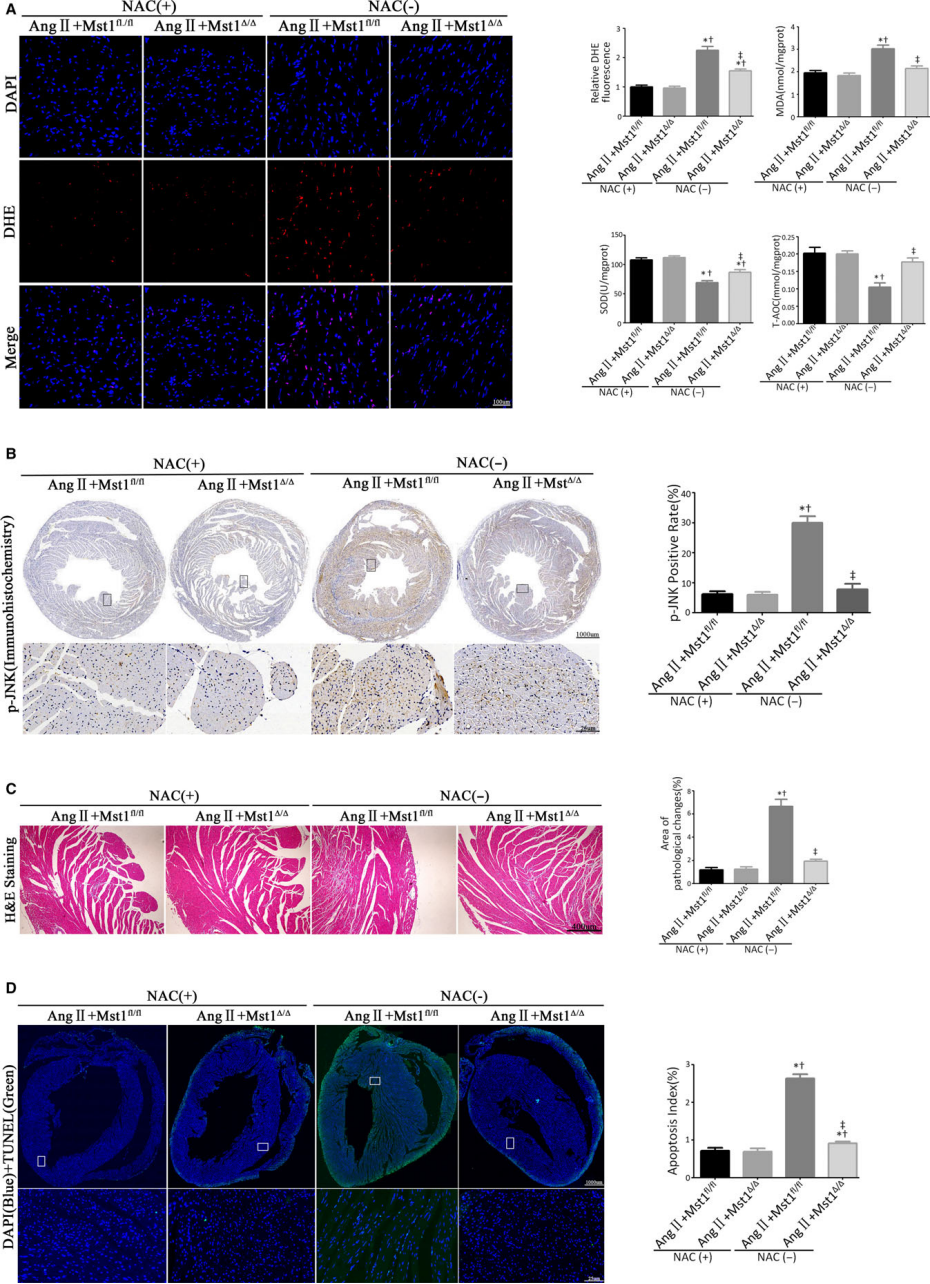
Mst1 deficiency inhibits ROS‐mediated JNK phosphorylation to attenuate Ang II‐induced cardiomyocyte apoptosis in vivo. A: Measurements of ROS production (n = 6). The effects of ROS generations were measured by the DHE fluorescent probe, the levels of intracellular MDA, SOD, and T‐AOC. Histogram: Relative DHE fluorescence intensity, MDA levels (nmol/mgprot), SOD levels (U/mgprot), and T‐AOC levels (mmol/mgprot). B: Representative images of p‐JNK immunohistochemistry (n = 6). Histogram: p‐JNK positive rate (%). C: Analyse myocardial pathological changes by H&E staining (n = 6). Histogram: Areas of pathological changes (%). D: Representative images of TUNEL assay (n = 6); TUNEL (green), DAPI (blue), and cTroponin I antibody (red). Histogram: Apoptosis index. *P <* *0.05 vs Ang II+Mst1^fl/fl^ +NAC (+) group; ^†^ *P *<* *0.05 vs Ang II+Mst1^Δ/Δ^ + NAC (+) group; ^&ddagger;^ *P *<* *0.05 vs Ang II+Mst1^fl/fl^ +NAC (−) group

To determine whether Mst1 deficiency suppresses JNK activation to alleviate Ang II‐induced cardiomyocyte apoptosis in an ROS‐dependent manner, mice were administered with the ROS scavenger NAC by intraperitoneal injection (Figure 3A). In Mst1^fl/fl^ mice with Ang II infusion, treatment with NAC abrogated the Ang II‐induced production of superoxide radical and suppressed Ang II‐triggered ROS production, as evidenced by the decreases in DHE fluorescence intensity, malondialdehyde (MDA) level, and total antioxidant capacity (T‐AOC) as well as the upregulation of superoxide dismutase (SOD). In NAC‐treated mice with chronic Ang II infusion, cardiomyocyte‐specific Mst1 knockout failed to further inhibit the production of active oxygen radicals (Figure 3A).

We also evaluated JNK phosphorylation by immunohistochemistry. In the presence of Ang II, NAC treatment reduced JNK phosphorylation in Mst1^fl/fl^ mice as compared to control mice (Figure 3B). Importantly, in Ang II infused mice with NAC treatment, cardiomyocyte‐specific Mst1 knockout failed to further decrease JNK phosphorylation level (Figure 3B).

Next, we carried out a histopathological analysis by hematoxylin and eosin stainings as well as determined the apoptotic index with TUNEL assay (Figure 3C and D). In NAC‐treated Mst1^fl/fl^ mice with Ang II infusion, NAC alleviated Ang II‐induced pathological changes and apoptosis (Figure 3C and D). Interestingly, there were no significant differences in these parameters between Mst1^fl/fl^ and Mst1^Δ/Δ^ mice treated with AngII and NAC (Figure 3C and D).

Taken together, these results indicate that the reversal of JNK phosphorylation by cardiomyocyte‐specific Mst1 knockout leading to the attenuation of Ang II‐induced cardiomyocyte apoptosis is dependent on the suppression of Ang II‐triggered ROS production.

### 3.5 Mst1‐specific knockout failed to reduce Ang II‐induced hypertension

There were no significant differences in MAP between all groups before chronic Ang II infusion. Six weeks later, the Ang II groups had higher MAP compared to the corresponding control groups (ESM Figure S5A‐B). However, there were no significant differences in MAP between the Ang II+Mst1^Δ/Δ^ mice and the Ang II+Mst1^fl/fl^ mice (ESM Figure S5A‐B).

### 3.6 Mst1 knockdown decreases Ang II‐induced cardiomyocyte apoptosis in vitro

**Figure 4:**
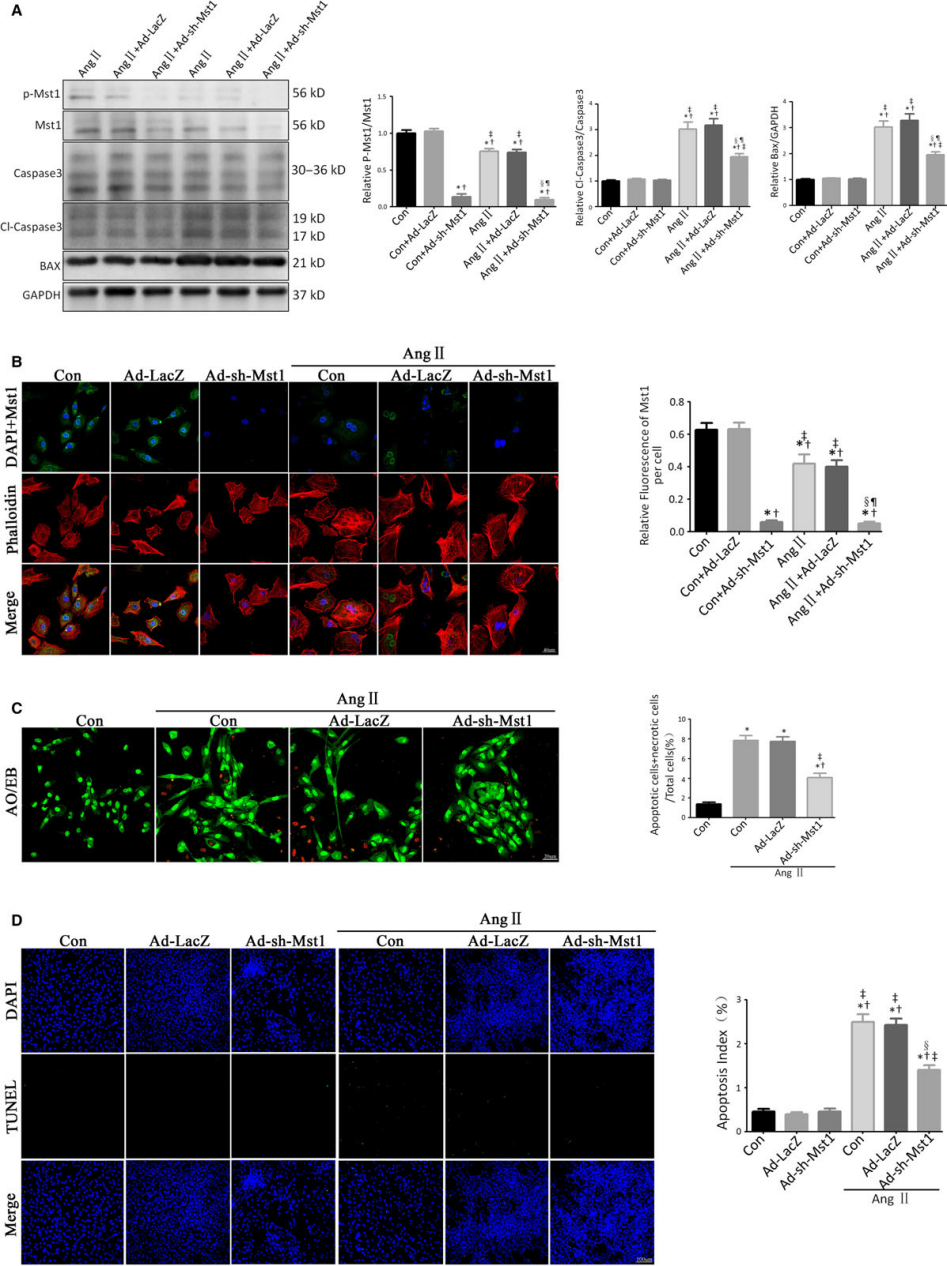
Mst1 knockdown decreases Ang II‐induced cardiomyocyte apoptosis in vitro. A: Immunoblots and quantitative analyses of p‐Mst1/Mst1, Cl‐caspase3/Caspase3 and Bax/GAPDH (n = 15). Histogram: Relative p‐Mst1/Mst1, Cl‐caspase3/Caspase3 and Bax/GAPDH. B: Representative images of Mst1 fluorescence detection (n = 15). Histogram: Realtive Fluorescence Intensity of Mst1 per cell. *P <* *0.05 vs Con group; ^†^ *P *<* *0.05 vs Con+Ad‐LacZ group; ^&ddagger;^ *P *<* *0.05 vs Con+Ad‐sh‐Mst1 group; ^§^ *P *<* *0.05 vs Ang II group; ^¶^ *P *<* *0.05 vs Ang II+Ad‐LacZ group. C: Representative images of AO/EB‐stained primary neonatal cardiomyocytes (n = 15). Histogram: Live apoptotic cells+necrotic cells (%). *P <* *0.05 vs Con group; ^†^ *P *<* *0.05 vs Ang II group; ^&ddagger;^ *P *<* *0.05 vs Ang II +Ad‐LacZ group. D: Representative images of TUNEL‐stained primary neonatal cardiomyocytes (n = 15). Histogram: apoptosis index (%). *P <* *0.05 vs Con group; ^†^ *P *<* *0.05 vs Ang II group; ^&ddagger;^ *P *<* *0.05 vs Ang II+ Ad‐LacZ group; ^§^ *P *<* *0.05 vs Ang II+Ad‐sh‐Mst1 group. In B‐D, 15 images were from different batches in different discs

Transduction of neonatal mouse cardiomyocytes with adenovirus harbouring short hairpin RNA against Mst1 abolished Mst1 expression (Figure 4A and B).

We evaluated the protein levels of cleaved caspase‐3 and Bax by western blotting and found similar trends in cleaved caspase‐3/caspase‐3 and Bax/glyceraldehyde 3‐phosphate dehydrogenase (GAPDH) ratios to those observed in vivo. Mst1 knockdown alleviated Ang II‐induced primary cardiomyocyte apoptosis, as evidenced by the downregulation of cleaved caspase‐3 and Bax in Ang II‐treated primary cardiomyocytes (Figure 4A).

The degree of cellular damage in cardiomyocytes was estimated by Acridine Orange/Ethidium Bromide (AO/EB) stainings. There were three types of primary cardiomyocyte after AO/EB stainings: live cardiomyocyte (appearing green), live apoptotic cardiomyocyte (appearing dark yellow), and necrotic cardiomyocyte (appearing red).^25^ The ratio of apoptotic cardiomyocytes+necrotic cardiomyocytes/total cardiomyocytes was increased by Ang II treatment (Figure 4C). Importantly, Mst1 knockdown attenuated Ang II‐induced primary cardiomyocyte damage, as evidenced by the decreased numbers of apoptotic cardiomyocytes+necrotic cardiomyocytes (Figure 4C).

Furthermore, the results of TUNEL assay showed that Ang II induced primary cardiomyocyte apoptosis while Mst1 knockdown abrogated this effect, consistent with in vivo findings (Figure 4D).

### 3.7 Mst1 knockdown reverses ROS‐dependent JNK activation to alleviate Ang II‐induced cardiomyocyte apoptosis in vitro

**Figure 5:**
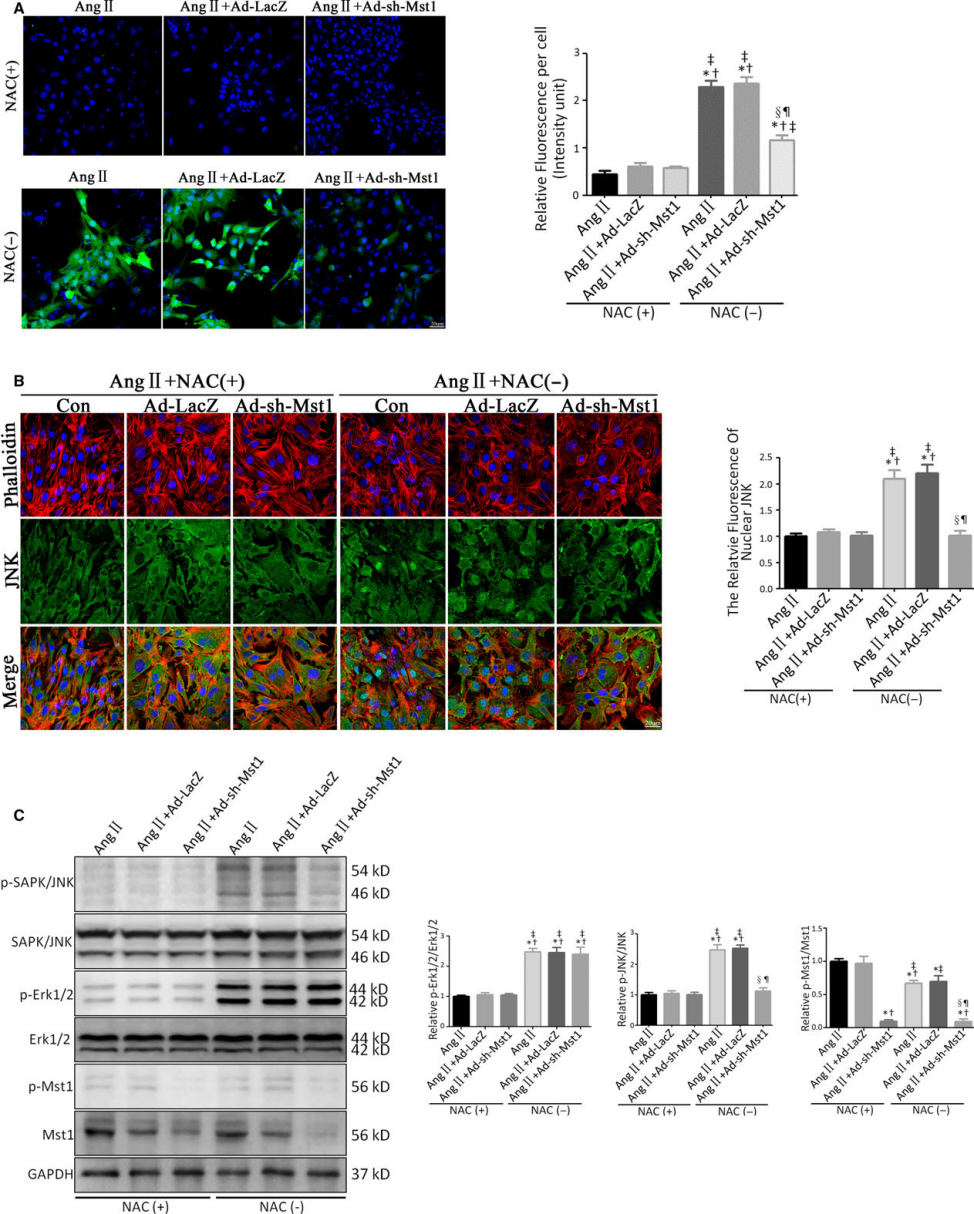
Mst1 knockdown reverses ROS‐dependent JNK activation to alleviate Ang II‐induced cardiomyocyte apoptosis in vitro. A: Representative images of DCFH‐DA probe‐labelled primary cardiomyocytes (n = 10). Histogram: Relative DCFH‐DA Fluorescence Intensity per cell. B: Representative images of JNK nuclear translocation indicated by immunofluorescence (n = 10). Histogram: The Relative Fluorescence of Nuclear JNK. In A‐B, 10 images were from different batches in different discs. C: Immunoblots and quantitative analyses of p‐Mst1/Mst1, p‐JNK/JNK and p‐Erk1/2/Erk1/2 (n = 10). Histogram: Relative intensity of p‐Mst1/Mst1, p‐JNK/JNK and p‐Erk1/2/Erk1/2. *P <* *0.05 vs Ang II+NAC (+) group; ^†^ *P *<* *0.05 vs Ang II+Ad‐LacZ+NAC (+) group; ^&ddagger;^ *P *<* *0.05 vs Ang II+Ad‐sh‐Mst1 + NAC (+) group; ^§^ *P *<* *0.05 vs Ang II+NAC (−) group; ^¶^ *P *<* *0.05 vs Ang II+Ad‐LacZ+NAC (−) group

To determine whether Mst1 knockdown suppresses intracellular ROS production in primary cardiomyocytes, we examined intracellular ROS levels using the fluorescent probe 2′, 7′‐dichlorofluorescin diacetate (DCFH‐DA). NAC treatment and Mst1 knockdown reversed the Ang II‐induced increase in intracellular ROS level (Figure 5A). Interestingly, Mst1 knockdown failed to further decrease ROS production following NAC treatment (Figure 5A).

To investigate the role of JNK signalling in Ang II‐induced cardiomyocyte apoptosis, we examined JNK nuclear translocation by immunofluorescence analysis and found that it was enhanced in the presence of Ang II (Figure 5B). This was reversed by NAC treatment or Mst1 knockdown (Figure 5B). Importantly, in Ang II‐treated primary cardiomyocytes, Mst1 knockdown failed to further suppress JNK nuclear translocation after NAC treatment (Figure 5, B).

We next evaluated the expression of apoptosis‐related proteins in primary cardiomyocytes by western blotting. Ang II‐induced the phosphorylation of JNK (Thr183/Tyr185) and ERK1/2, whereas NAC treatment abolished the activation of JNK and ERK1/2 (Figure 5C). Mst1 knockdown reversed JNK activation (Thr183/Tyr185) in the presence of Ang II but had no effect on ERK1/2 phosphorylation status, which is in accordance with the in vivo results (Figure 5C). Importantly, in Ang II‐treated primary cardiomyocytes, Mst1 knockdown did not further reverse JNK phosphorylation after NAC treatment (Figure 5C).

These results indicate that the protective effect of Mst1 deficiency against primary cardiomyocyte apoptosis caused by Ang II involves suppressing the generation of ROS.

### 3.8 Mst1 knockdown improves mitochondrial metabolism by inhibiting Ang II‐induced ROS production

Mst1 deficiency decreased Ang II‐induced intracellular ROS production, as seen by the increased levels of MDA and T‐AOC and downregulation of SOD (ESM Figure S6A‐C). Meanwhile, Mst1 deficiency enhanced mitochondrial metabolism (ESM Figure S6D).

In Ang II‐treated primary cardiomyocytes, loss of Mst1 did not further improve mitochondrial metabolism following treatment with NAC, indicating that Mst1 knockdown enhances mitochondrial metabolism by reversing Ang II‐induced oxidative stress (ESM Figure S6D).

### 3.9 Ang II‐induced JNK phosphorylation is reversed by Mst1 deficiency via enhanced binding of Trx to ASK1

**Figure 6:**
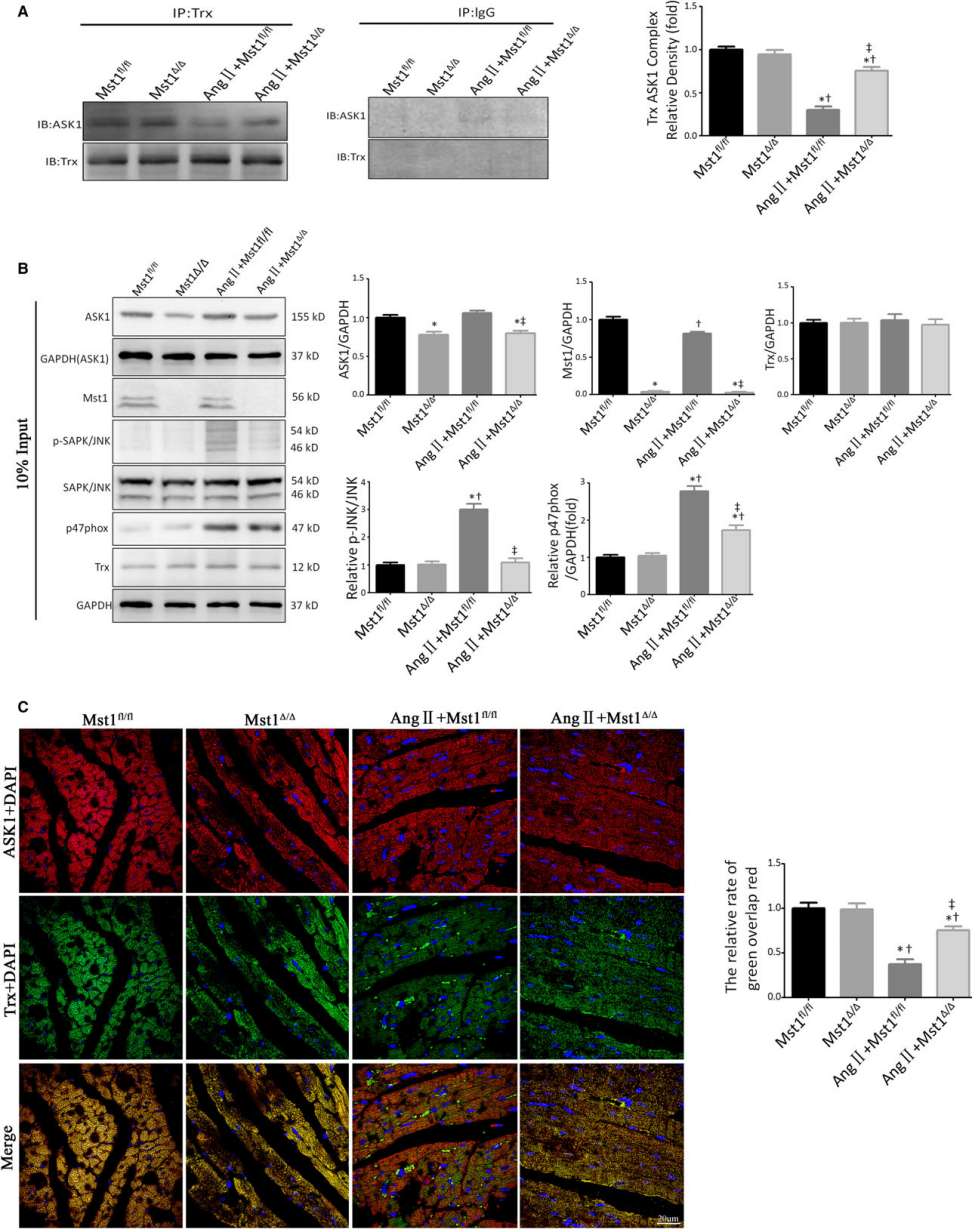
Ang II‐induced JNK phosphorylation is reversed by Mst1 deficiency via enhanced binding of Trx to ASK1. A: Trx/ASK1 protein binding in myocardial tissues at 7 weeks after chronic Ang II pump infusion (n = 9). Histogram: The relative intensity of Trx/ASK1 Complex. B: Immunoblots and quantitative analyses of Mst1/GAPDH, p‐JNK/JNK, ASK1/GAPDH, Trx/GAPDH and p47^phox^/GAPDH (n = 9). Histogram: Relative intensity of Mst1/GAPDH, p‐JNK/JNK, ASK1/GAPDH, Trx/GAPDH, and p47^phox^/GAPDH. C: Representative images of immunofluorescent double‐labelled co‐localisation of Trx and ASK1 (n = 9). Histogram: The relative ratio of green puncta (Trx) overlaps red puncta (ASK1). *P <* *0.05 vs Mst1^fl/fl^ group; ^†^ *P *<* *0.05 vs Mst1^Δ/Δ^ group; ^&ddagger;^ *P *<* *0.05 vs Ang II+ Mst1^fl/fl^ group

In the presence of Ang II, cardiomyocyte‐specific Mst1 knockout enhanced the interaction between Trx and ASK1, which suppressed ASK1 and downstream apoptotic signaling (Figure 6A). Meanwhile, Ang II+Mst^Δ/Δ^ mice showed markedly decreased JNK phosphorylation (Thr183/Tyr185) as well as ASK1 and p47^phox^ expression as compared to the AngII+Mst^fl/fl^ group (Figure 6B). Immunofluorescence double‐labelling experiments revealed greater co‐localization of Trx and ASK1 in the AngII+Mst^Δ/Δ^ mice as compared to the AngII+Mst^fl/fl^ group, indicating that loss of Mst1 promoted Trx and ASK1 binding (Figure 6C).

These results revealed that in the absence of Mst1, Trx and ASK1 combine to inhibit ASK1 activity, thus reversing ASK1/JNK‐mediated cardiomyocyte apoptosis induced by Ang II.

## 4 DISCUSSION

HF is a common cause of death among patients with cardiovascular diseases. Despite progress in our understanding of the molecular and cellular mechanisms that contribute to HF and the development of many therapies, the incidence of HF remains high.^1^

The major characteristics of heart failure include cardiomyocyte apoptosis and hypertrophy, which are largely the results of RAAS hyperactivation and the resultant production of ROS induced by the RAAS effector Ang II.^26^, ^27^, ^28^ Our previous study showed the role of cardiac‐specific Mst1 knockout in cardiac hypertrophy and cardiomyocyte autophagy; however, whether Mst1‐specific knockout involved in Ang II‐induced ROS production and cardiac apoptosis as well as the underlying mechanisms remain to be clarified.^29^ In this study, we showed that cardiomyocyte‐specific Mst1 knockout attenuates Ang II‐induced cardiomyocyte apoptosis by blocking JNK signalling, an effect that is dependent on ROS clearance. Cardiac‐specific Mst1 knockout also enhanced the binding of Trx to ASK1, thereby suppressing ASK1‐JNK signalling activation and inhibiting cardiomyocyte apoptosis. Interestingly, cardiac‐specific Mst1 knockout failed to reduce Ang II‐induced hypertension, which signifies that the role and mechanisms of Mst1‐specific deletion alleviating Ang II‐induced cardiomyocyte apoptosis were independent of the reduction of arterial pressure.

Some previous studies suggested that ROS induced cell apoptosis and NAC restored the ROS‐mediated apoptosis.^30^ However, Maiko Suzuki and colleagues reported that ROS also promotes antioxidant defense and plays a protective role in the posttranslational modification of proteins; NAC also induced apoptosis via mitochondrial‐dependent means.^31^ Therefore, the roles and underlying mechanisms of intracellular ROS and NAC in various cell types remain elusive.^31^ In this study, Ang II administration triggered the production of intracellular ROS, leading to cardiomyocyte apoptosis and cardiac dysfunction; this effect was abrogated by NAC and by cardiomyocyte‐specific Mst1 knockout, which resulted in the downregulation of p47^phox^, an NAD(P)H oxidase subunit. P47^phox^ is required for activation of the latent NADPH oxidase, a multicomponent enzyme that is activated to produce superoxide anion and ROS.^24^ Thus, Mst1 deficiency exerts anti‐apoptosis effect via inhibiting Ang II‐induced ROS production. This was found to be mediated via inhibiting activation of p47^phox^ and JNK signalling. Interestingly, the phenomenon that Ang II slightly decreased the level of p‐Mst1/Mst1 was observed in this study and our previous works, which could be interpreted as a host‐defense mechanism, a spontaneous repair of cardiomyocyte to try to attenuate the Ang II‐triggered oxidative stress and subsequent heart failure.^29^

MAPK pathways act as the downstream of ROS signalling under many pathological conditions.^32^ Increased phosphorylation of Mst1 (Thr183) was shown to activate upstream of MAPK kinases, sequentially altering the phosphorylation status of MAPKs.^32^ Our study suggested that cardiomyocyte‐specific Mst1 knockout abrogated the phosphorylation of Mst1 (Thr183), thus inhibiting the phosphorylation of JNK (Thr183/Tyr185); however, Mst1 deficiency failed to change Erk1/2 phosphorylation, suggesting that Mst1‐specific knockout had the specificity of MAPKs signalling transduction.

This study has indicated that JNK phosphorylation results from ROS‐induced apoptosis; conversely, suppressing JNK activation can block the initiation of apoptosis. Excess ROS production induced by Ang II can also perturb mitochondrial function; this was confirmed in the present study in primary cardiomyocyte, and was found to be reversed by Mst1 deficiency.

ASK1 is activated by ROS generated by mitochondria in the presence of Ang II.^33^, ^34^ Previous studies also showed that ASK1 relays apoptotic signals by directly phosphorylating JNK.^33^, ^34^ Trx, a highly conserved 12‐kDa protein, is involved in various physiological processes; inhibition of Trx was shown to promote cell apoptosis and induce the activation of ASK1 and downstream JNK signalling by unbinding from the N terminus ASK1, thereby enhancing ASK1 kinase activity by recruiting tumour necrosis factor receptor‐associated factor (TRAF)2 and TRAF6 to N‐terminal region of ASK1.^15^, ^22^, ^23^, ^34^ Our results showed that Ang II induced the dissociation of Trx from ASK1, leading to ASK1 activation and JNK phosphorylation. Importantly, under these conditions, loss of Mst1 enhanced the interaction between Trx and ASK1, resulting in the suppression of ASK1/JNK‐dependent apoptotic pathway.

A limitation in our study is the use of neonatal cardiomyocytes for in vitro experiments. Adult cardiomyocytes isolated from cardiomyocyte‐specific Mst1 knockout mice should be considered. Based on this, the potential binding sites between Mst1 and ASK1/Trx could be explored in future plan.

In conclusion, we demonstrate here that cardiac‐specific Mst1 knockout reduced Ang II‐triggered ROS production and alleviated cardiomyocyte apoptosis by counteracting the Ang II‐induced dissociation of Trx from ASK1 and thereby inhibiting ASK1/JNK‐mediated activation of apoptosis. These findings provide novel insight into the molecular link between the RAAS and cardiac apoptosis and suggest Mst1 as a potential therapeutic target for preventing HF.

## DUALITY OF INTEREST

The authors declare that there is no duality of interest associated with this manuscript.

## DISCLOSURES

None.

## AUTHOR CONTRIBUTION

Dongdong Sun, Haichang Wang and Zheng Cheng conceived the project, researched data, analyzed data and wrote the manuscript.

Xinyu Feng, Shanjie Wang, Mingming Zhang, Tingting Wang, Jie Lin, Erhe Gao and Jianqiang Hu researched and analysed the data.

All authors have revised the manuscript critically for important intellectual content and approved the final version to be published. Dongdong Sun is the guarantor of this work and, as such, had full access to all the data in the study and takes responsibility for the integrity and accuracy of the data analysis.
